# Telemedicine in Primary Practice in the Age of the COVID-19 Pandemic—Review

**DOI:** 10.3390/medicina59091541

**Published:** 2023-08-25

**Authors:** Anna Romaszko-Wojtowicz, Łukasz Jaśkiewicz, Paweł Jurczak, Anna Doboszyńska

**Affiliations:** 1 Department of Pulmonology, School of Public Health, Collegium Medicum, University of Warmia and Mazury in Olsztyn, 10-719 Olsztyn, Poland; anna.doboszynska@wp.pl; 2 Department of Human Physiology and Pathophysiology, School of Medicine, Collegium Medicum, University of Warmia and Mazury in Olsztyn, 10-082 Olsztyn, Poland; lukasz.jaskiewicz@uwm.edu.pl; 3 Student Scientific Club of Cardiopulmonology and Rare Diseases of the Respiratory System, School of Medicine, Collegium Medicum, University of Warmia and Mazury in Olsztyn, 10-082 Olsztyn, Poland; pawel.jurczak.1@student.uwm.edu.pl

**Keywords:** COVID-19, telemedicine, primary practice

## Abstract

*Background and Objectives*: In the era of the COVID-19 pandemic, telemedicine, so far underestimated, has gained in value. Currently, telemedicine is not only a telephone or chat consultation, but also the possibility of the remote recording of signals (such as ECG, saturation, and heart rate) or even remote auscultation of the lungs. The objective of this review article is to present a potential role for, and disseminate knowledge of, telemedicine during the COVID-19 pandemic. *Material and Methods*: In order to analyze the research material in accordance with PRISMA guidelines, a systematic search of the ScienceDirect, Web of Science, and PubMed databases was conducted. Out of the total number of 363 papers identified, 22 original articles were subjected to analysis. *Results*: This article presents the possibilities of remote patient registration, which contributes to an improvement in remote diagnostics and diagnoses. *Conclusions*: Telemedicine is, although not always and not by everyone, an accepted form of providing medical services. It cannot replace direct patient–doctor contact, but it can undoubtedly contribute to accelerating diagnoses and improving their quality at a distance.

## 1. Introduction

Telemedicine, also called telehealth, is the provision of health-related services over a distance using digital communication technologies. The origins of telemedicine date back to the 1950s, when the first mentions of the possibilities of the remote transmission of imaging tests appeared [1]. Telemedicine may take different forms, e.g., it can be in the form of communication between a patient and a doctor such as a doctor’s advice via telephone, chat, or videoconference. It may also be associated with more experimental innovations, e.g., telesurgery, where a surgeon remotely manipulates surgical instruments with the aid of a robotic surgical system.

The pandemic caused by the SARS-CoV-2 virus has stimulated the more rapid development of telemedicine. Pursuant to the WHO recommendations, telemedicine was chosen as a key strategy for the provision, maintenance, and enhancement of health-related services which were disrupted by the COVID-19 outbreak [2]. There are many scientific articles dedicated to the use of telemedicine in various specialties (e.g., internal diseases, family medicine, psychiatry, oncology) [3]. According to the report presented by Omboni et al., the most common purpose of using telemedicine (49.7%) was to ensure integrated patient care, including a combination of services with the aim of providing diagnosis, treatment, observation, and rehabilitation [4]. Monitoring patients in their home environment enables doctors to gain a better insight into the social conditions of their patients’ health statuses.

Of key importance for the development of telemedicine is the growing accessibility of technologies. In line with the Pew Research Centre’s report of 2021, 93% of Americans use the internet [5]. Moreover, 81% of Americans have smartphones, nearly 75% have desktops or laptops, and around 50% have tablets or e-readers [6]. Nowadays, telemedicine does not have to be limited to consulting one’s doctor by telephone, but can involve the use of other, more modern methods, e.g., measuring instruments which allow objectivization of symptoms reported by the patients and enable the doctor to make a correct diagnosis, which means that telemedicine can improve significantly. These are instruments such as cameras and video cameras in smartphones, digital stethoscopes, ophthalmoscopes, otoscopes, and various types of biosensors. This form of telemedicine, in which mobile medical devices and technologies are employed in order to collect health data generated by the patient (PGHD—patient-generated health data) and transmitted to health care providers, is referred to in the literature as remote patient monitoring (RPM) [7].

This starts from the simplest solutions, in which medical documentation is stored, monitored, and edited practically from anywhere in the world, to more complex ones related to artificial intelligence. In this way, it is possible, for example, to remotely supervise surgical procedures. Telemedicine is already widely used in emergency medicine systems. ECG transmission, in terms of qualification for invasive treatment, has already become a standard which is possible in most ambulances.

The purpose of this systematic review has been to evaluate telemedicine technologies that were employed during the COVID-19 pandemic, from the viewpoint of optimization of their use in situations of limited direct access to a physician. In particular, we are interested in the issue of using telemedicine techniques used in primary health care, as if creating a telemedicine primary care guide.

## 2. Material and Methods

The study was conducted according to the Preferred Reporting Items for Systematic Review and Meta-Analysis Protocols (PRISMA) statement [8]. The systematic review of the literature was based on an a priori definition of inclusion and exclusion criteria, which enabled an objective selection of articles dealing with the connections between telemedicine and COVID-19. This approach allowed us to preselect the research material objectively, preventing any subjectivity in our decisions to include some or exclude other studies from the review. The search employed the following terms: “telemedicine, telehealth, lung, respiratory, COVID-19, SARS-CoV-2, diagnosis, symptoms”.

The following search sequences were used in the work:

- “telemedicine”, “symptoms”, “diagnosis”, “covid-19”, “respiratory”, “lung”;
- “telehealth”, “symptoms”, “diagnosis”, “covid-19”, “respiratory”, “lung”;
- “telemedicine”, “symptoms”, “diagnosis”, “sars-cov-2”, “respiratory”, “lung”;
- “telehealth”, “symptoms”, “diagnosis”, “sars-cov-2”, “respiratory”, “lung”.

Due to the multitude of available studies and the symptomatology of COVID-19, the study was limited to searches related to the involvement of the respiratory system.

In this way, attempts were made to isolate publications enabling the assessment of the respiratory system, using methods of remote assessment of the general condition of patients, including remote registration. In particular, we wanted to present in the review solutions facilitating the work of a clinician at the level of primary care.

**Figure 1:**
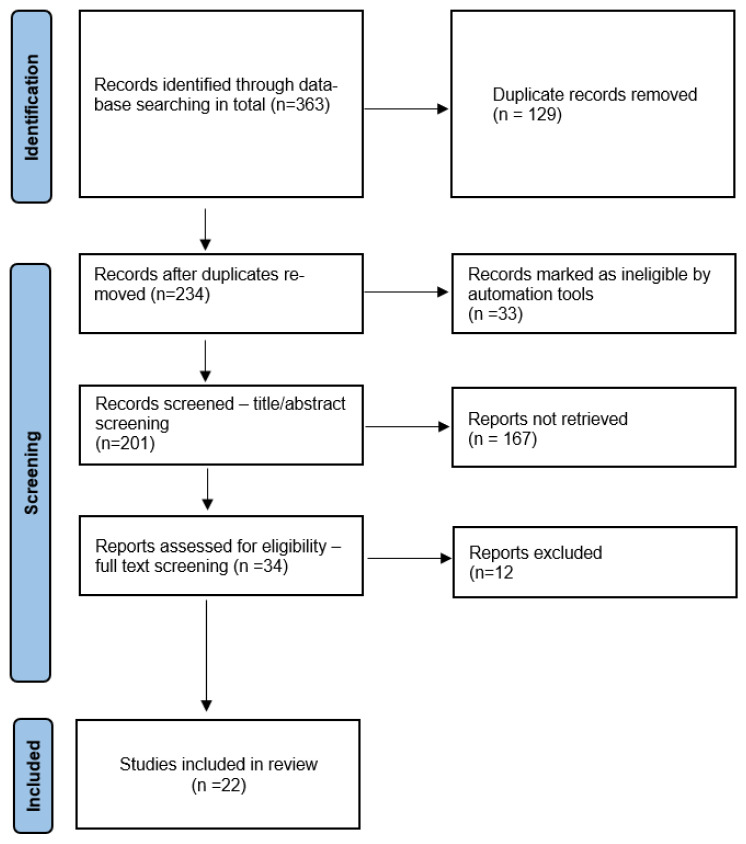
PRISMA flow diagram.

The review of the literature took advantage of the following databases: ScienceDirect, Web of Science, and PubMed. While searching the ScienceDirect database, a filter was activated to exclude meta-analyses, reviews (also systematic reviews), and books, in addition to which the search was limited to studies in medical sciences and that were original publications. The Web of Science database was searched for articles in medical sciences. As for PubMed, the same filters as applied to ScienceDirect were used. Some records were also retrieved via references found in published articles. No limits regarding the date of publication were set. Detailed results of the search are presented in a flow diagram (Figure 1) [9,10]. The search was carried out in the fourth and fifth weeks of March 2023.

### Selection of Studies and Exclusion Criteria

For further preselection of papers, the software Rayyan was employed [11]. Firstly, duplicates were eliminated and records implicated by the software as possibly being duplicates were reviewed. Next, three independent researchers analyzed the abstracts, and those dedicated to telemedicine methods used during the COVID-19 pandemic were selected. Lists of references were searched manually in order to identify further publications suitable for our analysis. Thus, articles were selected compliant with the previously established exclusion and inclusion criteria for a full-text review. Any disputes were resolved by consensus. In total, 234 publications were checked and 34 were chosen for complete analysis. Having analyzed the complete texts, 22 publications were selected for the systematic review.

**Figure 2:**
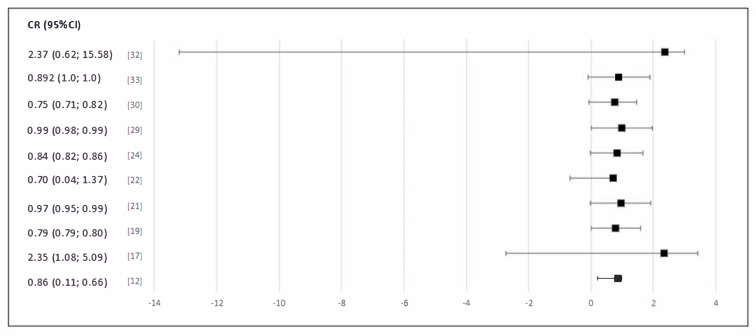
Plots of the proportion of the incidence of symptomatic and asymptomatic acute respiratory tract infection in COVID-19.

The inclusion criteria were to choose original articles, published in the English language, with clearly determined measures applied to clinical results. The exclusion criteria were to discard studies with unclear measures applied to clinical results, descriptions of single cases, or series of cases with a sample size <5. The articles selected for the final analysis are collated in Table 1. A forest plot was used to visually depict publication bias (Figure 2).

## 3. Results

**Figure 3:**
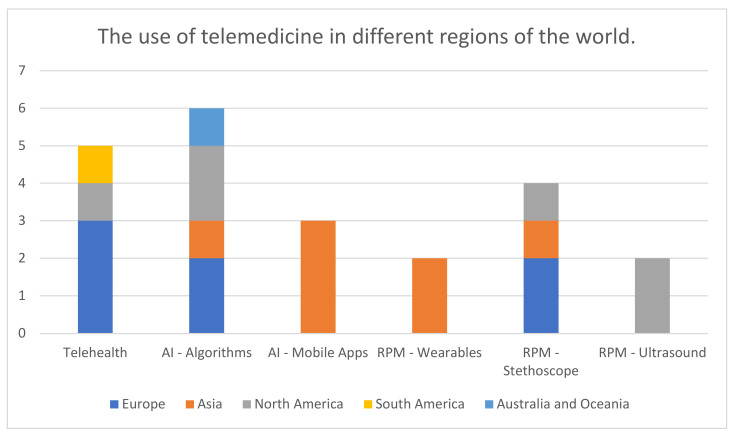
The use of telemedicine in different regions of the world. AI, artificial intelligence; RPM, remote patient monitoring.

The analyzed studies were published from July 2020 to December 2022. The 22 reviewed articles deal with seven issues, presented in Figure 2: telemedicine (telephone consultations, chats, and video consultations) (n = 5); AI techniques (n = 2) and algorithms (n = 4), that is, the issues related to telehealth and remote registration of patients, and the use of USG (n = 2); stethoscopes (n = 4); mobile applications (n = 3); and the “wearable body sensor network” (n = 2). Most studies were conducted in Europe (n = 7), and the remaining papers originated from Asia (n = 7), North America (n = 6), Australia (n = 1), and South America (n = 1). The most articles about telehealth covering various types of teleconsultations was published in Europe [13,14,15] and one work in North America [16] and South America [12]. In Asia, all publications concerned AI techniques and remote patient monitoring. It is worth noting that all publications on the use of mobile applications [23,24,25] and wearables [26,27] were created there. The use of stethoscopes as a tool for remote control of the patient’s health was established in Asia [29], Europe [28,30], and North America [31]. The articles on the use of ultrasonography in patient follow-up were exclusively from North America [32,33]. The creation of algorithms useful in medicine based on artificial intelligence has been described in Asia [21], Europe [19,20], North America [18,22], and Australia [17]. These divisions are presented in Figure 3.

**Figure 4:**
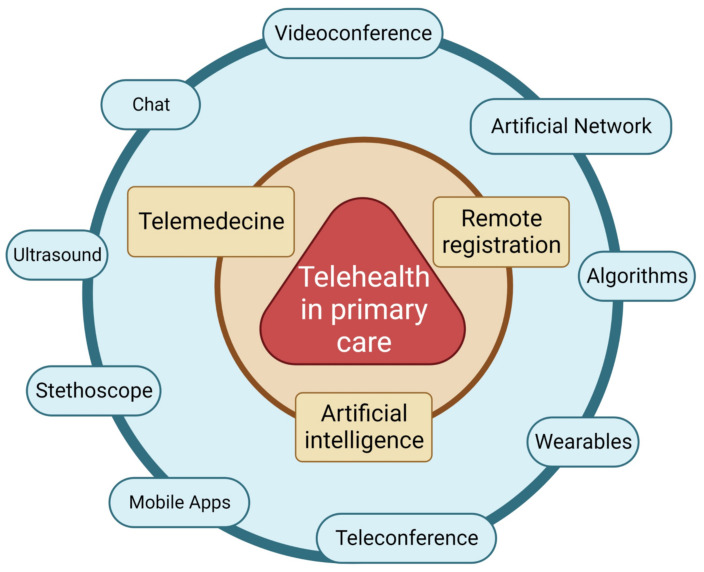
Telemedicine architecture used in the publication. Created with BioRender.com.

The preselected publications focused on the following aspects: providing health-related consultations via telemedicine using applications designed for this purpose, as well as a remote assessment of a patient’s condition with the help of the so-called wearable body sensor network, electronic stethoscopes, and a lung USG (Figure 4).

The presented review focuses on the aspects of telemedicine used in the practice of GPs. These methods focus on three main aspects, i.e., telehealth, methods of remote registration of patients, and the use of artificial intelligence methods for their assessment.

### 3.1. Telehealth

Telehealth consultations can be provided through various telemedicine channels, e.g., by telephone advice, chat, or videoconference. Remote consultations can be used both for a full patient interview, as well as to collect screening information enabling further proper triage of patients, which turned out to be particularly important during the COVID-19 pandemic [13]. The content and quality of consultations, regardless of their form (teleconsultation, chat, videoconsultation) are comparable [34].

### 3.2. Artificial Intelligence

Telemedicine solutions combined with artificial intelligence allow for the creation of algorithms that facilitate decision making or guide patients or doctors through the diagnostic path. These algorithms can be used to assess the general health status and cohort of patients. They can also make the first diagnosis based on the sent image of the throat or the recorded cough sound [21].

### 3.3. Remote Patient Monitoring

Remote patient registration methods are aimed at obtaining health parameters based on wearable body sensors, mobile apps, stethoscope, or ultrasound. Wearable body sensors, using sensors attached to the body, collect information about heart rate, saturation, respiratory rate, ECG, and body temperature [35]. It is also possible to remotely assess breath sounds and images of the lungs. Wearable body sensors are becoming an increasingly accurate diagnostic tool to help identify and treat diseases [36].

The detailed specifications of the articles, divided according to subject areas, is contained in Table 1.

## 4. Discussion

Telemedicine, also known as telehealth, involves the use of technologies to facilitate remote patient monitoring. Scientists have developed many different forms of telemedicine systems to fight the pandemic. This literature review is supposed to emphasize the possibilities of telemedicine methods which can be used in primary health care. The COVID-19 pandemic itself led to a significant increase in the number of health-related consultations provided with the help of ICT tools. This has necessitated, in a certain manner, the development of a collaboration between IT and medicine for the best possible objectivization of the health services provided. Monitoring the health status of patients with COVID-19 remotely can take different forms, from giving advice by telephone to using custom-designed teleinformation tools via the internet. Appropriate remote monitoring of the health of patients and chronic conditions can help to reduce the number of patients who need to be hospitalized, and this lowers the costs of medical care [37]. In a paper published in 2023 in JAMA, it was shown that telemedicine advice contributes to significant time and cost savings, including transport costs and the cost of medical visits. Increasing access to a doctor results in a reduction in the number of visits, hospitalizations, and mortality [38].

In our study, we have shown the possible division of telemedicine into three categories: telehealth, AI, and remote patient monitoring. This division has been used in order to easily present the possibilities of remote medical care methods, e.g., in a family doctor’s office. Thanks to the extremely rapid progress of technology in the 21st century, medical care provided through telemedicine channels does not have to differ much from direct contact with the patient. Not only that, according to the data presented in Figure 2, the recognition of respiratory tract infections by various methods of telemedicine can be just as effective.

### 4.1. Telehealth

During the outbreak of the COVID-19 pandemic, teleconsultations served to provide adequate education to patients in order to restrain the spread of the disease. Furthermore, telemedicine gave patients emotional support [13]. However, it soon turned out that teleconsultations were on the front line in effective management and conduct during the COVID-19 pandemic. Telephone history taking in primary health care was effective in detecting pneumonia in patients diagnosed with mild SARS-CoV-2 [16]. Accorsi et al., in their randomized trial, showed that teleconsultation is comparable to an in-patient consultation for patients with a low risk of progressing to a severe course of COVID-19 infection who developed symptoms of acute respiratory tract infection [12].

Truong et al., based on their systematic review, confirmed that teleconsultations show a high level of care and satisfaction of patients [39].

### 4.2. Artificial Intelligence

An artificial neural network (ANN), or simply a neural network, is a method of supervised learning. The learning process tries to mimic the learning that takes place in the human brain. Artificial intelligence models are employed to create appropriate groups of patients [23]. AI enables researchers to use data collected, for example, in the form of digital diagnostic tests, in order to improve telemedicine consultations.

Artificial intelligence methods based on a convolutional neural network (CNN) were used to evaluate chest X-rays of patients with COVID-19. [25] They served to help diagnose and classify COVID-19 cases, and to distinguish patients with COVID-19 from other patients with (viral or bacterial) pneumonia [40,41,42,43]. It was demonstrated that a preliminary evaluation of chest X-rays could be achieved remotely, considerably limiting of person-to-person contact. A deep learning model (CycleGAN) was used by Yoo et al. to detect severe cases of pharyngitis using a smartphone [21]. This approach was employed for screening patients in order to rapidly identify cases of pharyngitis and launch proper diagnostic and treatment procedures.

Algorithms created by artificial intelligence can support the diagnostic and therapeutic process. An example of this can be the management of community-acquired pneumonia, which can be one of the manifestations of COVID-19. Diagnosis of community-acquired pneumonia (CAP) is based on an evaluation of the signs and symptoms of a respiratory tract infection. Its clinical image varies. The manifestations of pneumonia can be divided into two groups: systemic (pyrexia, chills, malaise) and related to the respiratory system (cough, dyspnea, chest pain). The diagnosis of pneumonia in outpatients can be made on the basis of clinical manifestation not necessarily confirmed by laboratory and imaging tests. This option gained particular importance during the COVID-19 pandemic when the isolation of patients and the limitation of direct human contact were most important from the point of view of epidemiology. Porter et al. proved that the mathematical algorithm they tested enabled the accurate identification of patients with CAP of varying severity, excluding an analysis of vital signs and physical and radiological examinations, in addition to which it ensured an immediate result. These Australian researchers built an algorithm on the basis of such symptoms as pyrexia, acute cough <7 days (registered by a smartphone), and age. The algorithm was then used to make a preliminary selection of patients [17].

Coronado-Vázquez et al. carried out telemedicine monitoring of patients with COVID-19, which enabled early detection of complications as well as the monitoring and treatment of concomitant illnesses, thereby contributing to reducing the risk of hospitalization [15]. In turn, Liu et al., who used an online application specially developed for their study, enabled patients to provide in real time all new data regarding the course of the illness, which contributed to gaining a better insight into the disease itself and improved overall evaluation of the health status of a given patient. The above researchers conducted medical consultations using an online application, including a preliminary triage, with the help of a voice conversation, text messaging, photo communication, or a video call. Out of 4589 patients, 310 were referred to the hospital and 301 were recommended to see a doctor in an in-patient setting (e.g., in a hospital) for physical examination. The cited authors demonstrated that telemedicine can facilitate the initial selection of patients, particularly during health crises such as COVID-19 [23].

Telemedicine services provided via another application (nCapp) were presented by Yang et al. [24]. In this case, the mobile tool used served to synchronize and share the data concerning the diagnosis and previous treatment. The aim of this application was to enable early diagnosis of COVID-19 and classification of patients (with a focus on patients with ambiguous or false negative results of RT-PCR tests) to appropriate risk groups. Owing to this application, the remote management of new cases of COVID-19 infections was improved.

Amjad et al., in their systematic review published in January 2023, have summarized the methods of artificial intelligence currently used in medicine. They proved that telehealth based on artificial intelligence can lead to an improvement in the quality of medical practice and also contribute to its modernization [44].

### 4.3. Remote Patient Monitoring

Monitoring of the health conditions of patients may take different forms, and one option employed nowadays is to use biosensors, which enable making non-invasive measurements. The sensors used in these devices read parameters from the skin or from the movements made by the person being monitored [45,46]. This allows the monitoring of a patient in any circumstances, including at home, remotely. The wearable body sensor network, according to Qureshi et al., is most probably the best solution for the remote monitoring of patients in health care systems. Such sensors as accelerometers, temperature sensors, or ECGs collect information about the health of a patient, acting as a monitoring network. The data are stored in a local server and can be retrieved by a clinician to aid the decision-making process [47].

Bassam et al. described a system that makes use of an online application as an external interface and an Android-based mobile application for the patient [27]. Both interfaces are synchronized with each other in order to gather data on the health of a patient. The system is composed of a device mounted like a bracelet on one of the patient’s limbs. It allows recording of the following parameters: body temperature, systolic heart rate, saturation, and cough episodes. It also has a built-in GPS reader.

Similar systems used during the COVID-19 pandemic for remote registration of vital signs have been presented in the literature by Balasubramanian V., Ding X, Romaszko et al. [26,48,49].

It seems that wearable body sensors may solve the monitoring problem, as was shown by Snehi et al. [50].

Telemedicine can play a role in decreasing the costs of health care borne by the patient and by the health care system. Adequate remote monitoring of health conditions and chronic conditions can help patients to avoid expensive visits to hospital emergency wards and even hospitalization. A stethoscope is an inexpensive instrument that is easy to use, but which can considerably facilitate making a preliminary diagnosis. However, its usefulness largely depends on the user’s perceptual ability and experience. In recent years, there has been a growing interest in the automation of auscultation, its standardization and digitalization. This trend gained momentum during the COVID-19 pandemic when any form of a remote physical examination helped to make a diagnosis via telemedicine. A team of researchers from Switzerland created an algorithm for the diagnosis and stratification of COVID-19 risk based on lung auscultation [28]. To this aim, an algorithm involving artificial intelligence and deep learning was developed that achieved standardization of lung auscultation. The data for their research were recorded with a digital stethoscope Littmann 3200. It was demonstrated that automated interpretation of lung auscultation can help to improve the accuracy of a physical examination. In turn, Zhu et al. employed artificial intelligence models and showed that an AI-based system can identify correctly different types of irregular murmurs detected during an auscultation examination of the lungs [29].

Pancaldi et al. created an algorithm called VECTOR (Velcro crackles detector) to evaluate Velcro crackles registered with the help of a digital stethoscope [51]. In 2022, these authors used the VECTOR algorithm to identify patients with interstitial pneumonia secondary to SARS-CoV-2 infection [30]. To this aim, a digital stethoscope Littmann 3200 was used to evaluate respiratory crackles in eight auscultation points, i.e., paravertebral lower lobes, axillary lower lobes, paravertebral middle lobes, and paravertebral upper lobes. The automated lung auscultation results proved the potential usefulness of artificial intelligence methods in the near future.

Undoubtedly, the COVID-19 pandemic has contributed to the search for new methods of remote diagnosis. In March 2023, *Diagnostics* published a paper comparing the types of stethoscopes, including electronic stethoscopes. The authors of this article showed many benefits of remote lung auscultation, or even the sound recording itself. They also did not omit the problem related to the use of artificial intelligence to interpret the recorded auscultation phenomena, which primarily result from their possible variability [52].

A group of 27 volunteers was submitted to a study in which a portable USG device and a teleconference on the Zoom platform were used to examine the images of the lungs of patients with COVID-19 [32]. The purpose of the study was to generate an adequate “batwing” image of the pleura interface between two rib shadows at each location on the thorax. The use of ultrasound ensures reliable monitoring and stratification of the risk of developing serious illnesses. These conclusions were confirmed by Kimura et al., in a study including 201 patients, in which the presence of a B line, which is associated with a higher risk of hospitalization of high-risk patients, was detected in the early phase of SARS-CoV-2 infection [33]. Moreover, these researchers confirmed that patients were able to perform a simple lung USG test themselves. Heldeweg et al., based on a systematic review of the literature, proved that ultrasound imaging of the lungs significantly affects clinical decision making, especially in the so-called places for quick diagnostics (e.g., emergency departments) [52].

The usefulness of a remote ultrasound exam has also been verified in cases of abdominal USG tests [53]. However, because of the topic area of this study not pertaining to the subject of our review, this paper was not included in the current systematic review.

Telemedicine is a rapidly developing field of medicine which has already been used widely owing to its usefulness and ability to provide medical services safely during the COVID-19 pandemic. It is obvious that telemedicine, nowadays, cannot replace personal patient care and that not all clinical situations can rely on video consultations alone, but it is also undeniable that in the time of medical staff shortages, telemedicine can help to improve the monitoring of the health status of patients, the functionality of the health care system, as well as the accessibility to medical advice. Not long ago, the only option for remote communication was by letter. Other remote communication channels, not to mention remote medical diagnosis methods, were unknown. The COVID-19 pandemic showed that medical care in the form of telehealth could be the not-so-distant future of medicine. Technological progress, a collaboration between IT and medicine, and the development of pro-health applications, such as ‘Apple-health’, remote auscultation, or imaging of internal organs, may become commonplace in everyday life.

The variety of methods used in telemedicine allows for almost unlimited possibilities for patients’ remote registration. At present, it is possible to conduct a full history-taking using remote methods of communication as well as remote registration and transmission of vital measurements enriched with the possibility of recording auscultatory changes. The analysis of these data using artificial intelligence methods minimizes the risk of medical error.

## 5. Conclusions

The COVID-19 pandemic has changed the perception of telemedicine. Conducted scientific studies have shown that telemedicine can be highly effective in recognizing upper respiratory tract infections, regardless of the type of method used.

The rapid development of 21st century technology may soon lead to even more efficient methods of telemedicine.

## Figures and Tables

**Table 1:** Detailed breakdown of work selected for analysis.

Authors (Ref.)	Number of Patients	Country	Study Design	Publication Date	Journal
Telehealth					
1. Teleconsultations					
Accorsi TAD, et al. [12]	98	Brazil	Randomized controlled trial	May 2022	*Einstein (Sao Paulo)*
Zhang A, et al. [13]	416	Netherlands	Descriptive survey—Case reports	Mar 2022	*Healthc (Amst).*
Baena-Díez JM, et al. [14]	453	Spain	Cohort study	Nov 2021	*Healthcare (Basel)*
Coronado-Vázquez V, et al. [15]	166	Spain	Cohort study	May 2021	*J. Pers. Med.*
Khairat S, et al. [16]	1139	USA	Cross-sectional—descriptive study	Oct 2020	*J. Patient Exp.*
Artificial intelligence					
2. Algorithms					
Porter P, et al. [17]	322	Australia	Cohort study	Mar 2021	*Br J Gen Pract.*
Jose T, et al. [18]	765,324	USA	Descriptive survey	Feb 2021	*Mayo Clin Proc Innov Qual Outcomes*
Eythorsson E, et al. [19]	4756	Iceland	Cohort study	Sep 2022	*Diagn Progn Res.*
Tartaglia E, et al. [20]	200–875/day	Italy	Observational study	Dec 2022	*Smart Health (Amst).*
Yoo TK, et al. [21]	339	South Korea	Cross-sectional—analytical study	Oct 2020	*Comput Biol Med.*
Li H, et al. [22]	965	USA, China	Cross-sectional—analytical study	Dec 2022	*Smart Health (Amst).*
3. Mobile Apps					
Liu L, et al. [23]	4589	China	Cohort study	July 2020	*JMIR Mhealth Uhealth.*
Yang D, et al. [24]	3249	China	Multi-center clinical study	Dec 2022	*Clinical eHealth*
Ahmad M, et al. [25]	185	India	Cross-sectional—analytical study	June 2022	*Diabetes Metab Syndr.*
Remote Patient Monitoring					
4. Wearable Body Sensors					
Balasubramanian V, et al. [26]	1200	India	Cross-sectional—analytical study	Jan 2022	*Med Biol Eng Comput.*
Al Bassam N, et al. [27]	-	Oman	Cross-sectional—descriptive study	May 2021	*Inform Med Unlocked.*
5. Stethoscope					
Glangetas A, et al. [28]	1000	Switzerland	Cohort study	Mar 2021	*BMC Pulm Med.*
Zhu H, et al. [29]	172	China	Cohort study	Jan 2022	*Computer Methods and Programs in Biomedicine*
Pancaldi F, et al. [30]	28	Italy	Cross-sectional—analytical study	Mar 2022	*Comput Biol Med.*
Lalouani W, et al. [31]	128	USA	Descriptive survey	Dec 2022	*Smart Health (Amst).*
6. Ultrasound					
Kirkpatrick AW, et al. [32]	27	Canada, USA	Randomized controlled trial	Dec 2022	*Ultrasound J.*
Kimura BJ, et al. [33]	201	USA	Cross-sectional—analytical study	Oct 2022	*J Am Soc Echocardiogr.*

## Data Availability

The data are available in a publicly accessible repository.
